# Ultrasound evaluation of the airway in the ED: a feasibility study

**DOI:** 10.1186/s13089-018-0083-6

**Published:** 2018-01-18

**Authors:** Elizabeth A. Hall, Ibrahim Showaihi, Frances S. Shofer, Nova L. Panebianco, Anthony J. Dean

**Affiliations:** 1 Department of Emergency Ultrasound, Emergency Medicine, Hospital at the University of Pennsylvania, Philadelphia, PA USA; 2 Ashtabula, OH 44004 USA; 3 Department of Emergency Ultrasound, Hospital at the University of Pennsylvania, Philadelphia, PA USA; 4 Epidemiology & Biostatistics, Department of Emergency Medicine, Hospital at the University of Pennsylvania, Philadelphia, PA USA; 5 Division of Emergency Ultrasonography, Hospital at the University of Pennsylvania, Philadelphia, PA USA

**Keywords:** Point-of-care ultrasound, Upper airway ultrasound, Airway management

## Abstract

**Background:**

Recognition of the difficult airway is a critical element of emergency practice. Mallampati score and body mass index (BMI) are not always predictive and they may be unavailable in critically ill patients. Ultrasonography provides high-resolution images that are rapidly obtainable, mobile, and non-invasive. Studies have shown correlation of ultrasound measurements with difficult laryngoscopy; however, none have been performed in the Emergency Department (ED) using a consistent scanning protocol.

**Objectives:**

This study seeks to determine the feasibility of ultrasound measurements of the upper airway performed in the ED by emergency physicians, the inter-rater reliability of such measurements, and their relationship with Mallampati score and BMI.

**Methods:**

A convenience sample of volunteer ED patients and healthy volunteers with no known airway issues, aged > 18 years, had images taken of their airway using a standardized ultrasound scanning protocol by two EM ultrasound fellowship trained physicians. Measurements consisted of tongue base, tongue base-to-skin, epiglottic width and thickness, and pre-epiglottic space. Mean and standard deviation (SD) were used to summarize measurements. Inter-rater reliability was assessed by intraclass correlation coefficients (ICCs). Analysis of variance with linear contrasts was used to compare measurements with Mallampati scores and linear regression with BMI.

**Results:**

Of 39 participants, 50% were female, 50% white, 42% black, with median age 32.5 years (range 19–90), and BMI 26.0 (range 19–47). Mean ± SD for each measurement (mm) was as follows: tongue base (44.6 ± 5.1), tongue base-to-skin (60.9 ± 5.3), epiglottic width (15.0 ± 2.8) and thickness (2.0 ± 0.37), and pre-epiglottic space (11.4 ± 2.4). ICCs ranged from 0.76 to 0.88 for all measurements except epiglottis thickness (ICC = 0.57). Tongue base and tongue base-to-skin thickness were found to increase with increasing Mallampati score (*p* = .04, .01), whereas only tongue-to-skin thickness was loosely correlated with BMI (*r* = .38).

**Conclusions:**

A standardized ultrasound scanning protocol demonstrates that the airway can be measured by emergency sonologists with good inter-operator reliability in all but epiglottic thickness. The measurements correlate with Mallampati score but not with BMI. Future investigation might focus on ultrasound evaluation of the airway in patients receiving airway management to determine whether ultrasound can predict challenging or abnormal airway anatomy prior to laryngoscopy.

## Background

Recognizing difficult airways in the Emergency Department (ED) is an important skill anatomic variations in airway structures cannot be fully assessed prior to intubation. Identification of a difficult airway prior to intubation allows for optimal preparation, equipment selection, and participation of experienced personnel.

Pre-intubation evaluation of the airway has traditionally depended on clinical parameters such as body mass index, neck circumference, and the Mallampati scoring method [1]. None of these parameters is reliably predictive [2–5]. A meta-analysis of 55 studies identified that only 35% of difficult intubations had a Mallampati score of III or IV [6]. Thus, it is clear that airway evaluation prior to laryngoscopy might benefit from improved methods of assessment.

Ultrasound is a mobile, non-ionizing, non-invasive tool that readily provides images of airway anatomy. Research in the anesthesiology literature has demonstrated that ultrasound can visualize key anatomical structures through transcutaneous views of the neck [7–10].

Studies examining the ability of ultrasound to predict difficult airways have been performed in adult patients in the pre-operative surgical setting using variable patient positioning and scanning protocols. Ultrasound measurements at the level of tongue, pharynx, larynx, and trachea have been compared to Cormack–Lehane Grades (CLG) determined during direct laryngoscopy [11]. Difficult laryngoscopy was found to correlate with ultrasound measurements taken at the hyoid bone, thyrohyoid membranes, and hyomental distance with patients in the sniffing position [12–14]. A South Korean study used ultrasound to examine the epiglottis in normal patients and those diagnosed with acute epiglottis and found a significant difference in the anteroposterior diameter, promoting ultrasound as a possible tool for diagnosing acute epiglottis [15]. Despite these results, several studies have had contradictory findings, and all were limited by small sample size, marked differences in population characteristics, and an absence of a specified ultrasound scanning protocol [16].

The current study investigates the feasibility of soft tissue measurements performed by clinician sonologists in the ED environment using a defined scanning protocol. The goal of this study was (1) to determine the inter-rater reliability of airway measurements obtained by emergency sonologists using a standardized scanning protocol and (2) to compare these measurements to widely used clinical predictors of difficult airway, specifically the Mallampati score and BMI.

## Methods

### Study design

This was a prospective study, using a convenience sample of ED patients and volunteer members of the ED staff. Airway ultrasound measurements were obtained by two emergency medicine (EM) physicians using a standardized scanning protocol. Both physicians were currently enrolled in an emergency ultrasound fellowship program. Mean and standard deviation (SD) were used to summarize measurements. The results obtained by the two sonologists were compared. Inter-rater reliability between the two sonologists was assessed by intraclass correlation coefficients (ICCs). Analysis of variance with linear contrasts was used to compare measurements with Mallampati scores and linear regression with body mass index (BMI).

### Study setting and population

The study was performed in a metropolitan tertiary care center with an annual ED census of 72,000 patients. The ED supports an Emergency Medicine Residency Program and an Emergency Ultrasound Fellowship. Subjects were ED patients presenting for complaints unrelated to the airway. They were asked to enroll voluntarily with the understanding that the ultrasound would likely have no impact on their clinical care. The study was approved by the institutional review board of the Hospital at the University of Pennsylvania.

### Selection of participants

We enrolled a convenience sample of patients and volunteers between March 2016 and September 2016. Patient inclusion criteria included age older than 17 years and ability to provide written consent in English. Volunteers were excluded if they had a history of congenital or acquired airway abnormalities.

### Study protocol

Any ED patient or ED staff volunteer that met inclusion criteria was approached by the research physicians for enrollment in the study and a written informed consent was obtained. Subjects had ultrasound measurements and images of their tongue base, tongue base-to-skin, epiglottic width and thickness, and pre-epiglottic space recorded separately by both physicians. The data were collected at bedside and each research physician was blinded to the other’s assessment. Demographic information including sex, age, race, BMI, and Mallampati scores were collected.

### Ultrasound technique

**Fig. 1:**
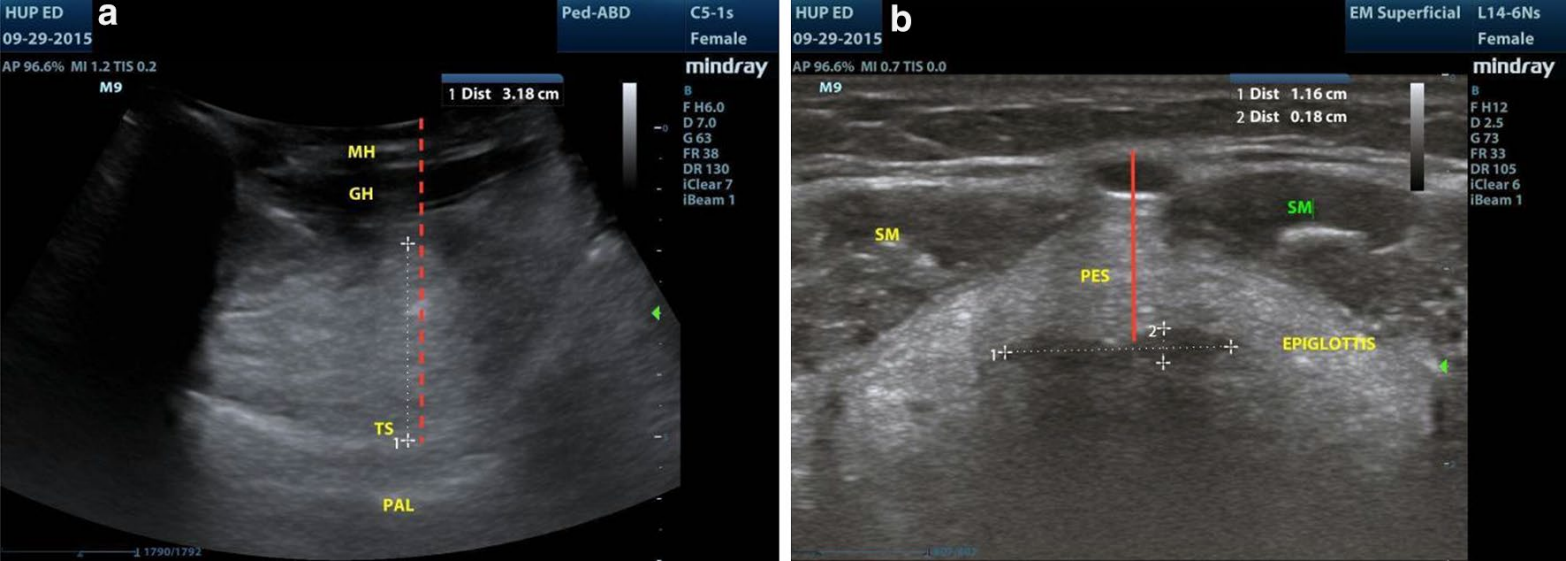
**a** Sagittal paramedian view of the tongue from the submental area with the white dotted line representing the tongue base measurement and the red dotted line as tongue base-to-skin thickness. The patient’s face would be in the direction of the right of the screen. *MH* mylohyoid muscle, *GH* geniohyoid muscle, *TS* tongue surface, *PAL* palette. **b** Transverse ultrasound image at the level of the thyrohyoid membrane. The pre-epiglottic space (red line, PES) is measured from the anterior surface of the epiglottis to the anterior margin of the strap muscles (SM) midway between the hyoid bone and the thyroid cartilage. The epiglottis is indicated by the two white caliper lines showing width (dotted line #1) and thickness (dotted line #2)

A standardized scanning protocol was used for subject positioning and measurement. Subjects were placed in the supine position without a pillow, with head and neck in extension, and tongue in resting position and touching the lower incisors. A Mindray M9 ultrasound machine (Mindray Bio-Medical Electronics Co, Shenzhen, China) with a curved linear 5- to 3-MHZ probe was used to obtain the tongue base and tongue base-to-skin thickness. A linear 10- to 5-MHZ probe was used to obtain the epiglottic width and thickness, and pre-epiglottic space measurements. All ultrasound images were obtained using the minimal transducer pressure necessary to obtain good skin contact. Subjects were asked to rest the tip of their tongue against their lower incisors while the measurements were being made. The curvilinear probe was placed in the longitudinal position or long axis along the submental space and the maximum thickness of the tongue was recorded as well as the maximum thickness of the tongue to the skin surface at the neck (Fig. 1a). The linear probe in a transverse plane was used to measure the epiglottic width and thickness at the level of the thyrohyoid membrane midway between the hyoid bone and the thyroid cartilage (Fig. 1b). The epiglottis was identified at the thyrohyoid membrane by making slight cranial or caudal angulated movements with the transducer and observing the bright air–mucosa interface at the posterior edge of the epiglottis. The epiglottis thickness was measured at the point of greatest visible thickness determined by the sonologist. At the same level, the pre-epiglottic space was measured from the anterior surface of the epiglottis to the anterior surface of the strap muscles (Fig. 1b). Demographic variables and Mallampati scores were collected by the same co-investigators after ultrasound measurements had been completed.

## Results

**Fig. 2:**
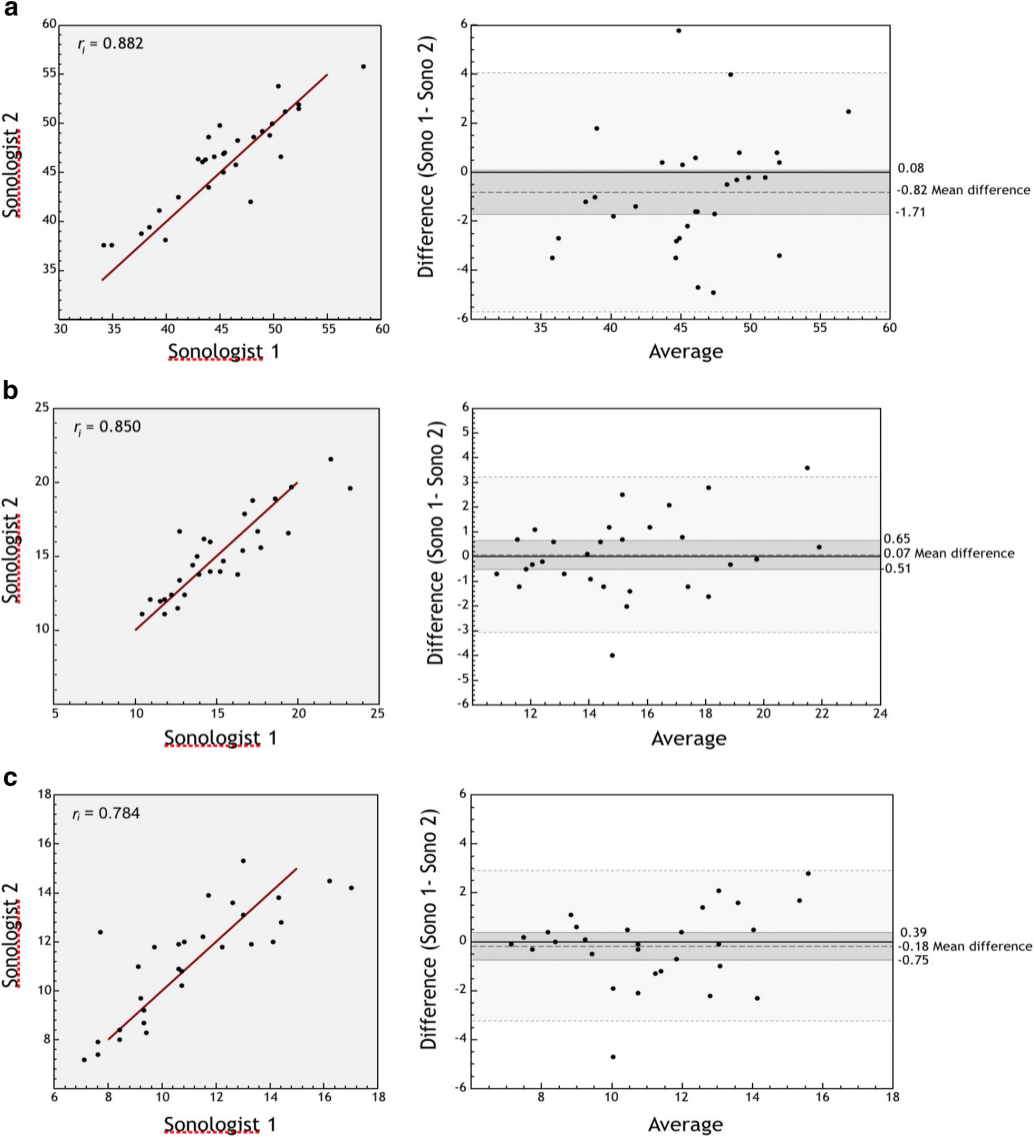
ICC graphs and Bland–Altman plots: agreement between sonologists in measurements. *Y* axis for Bland–Altman plots represents average of sonologists measurements. Mean difference between sonologists with 95% confidence intervals on the means shown (darker gray). Lighter gray bands represent the 95% confidence bands. **a** Tongue base thickness (mm). **b** Epiglottis LA (mm). **c** Pre-epiglotic space (mm)

**Fig. 3:**
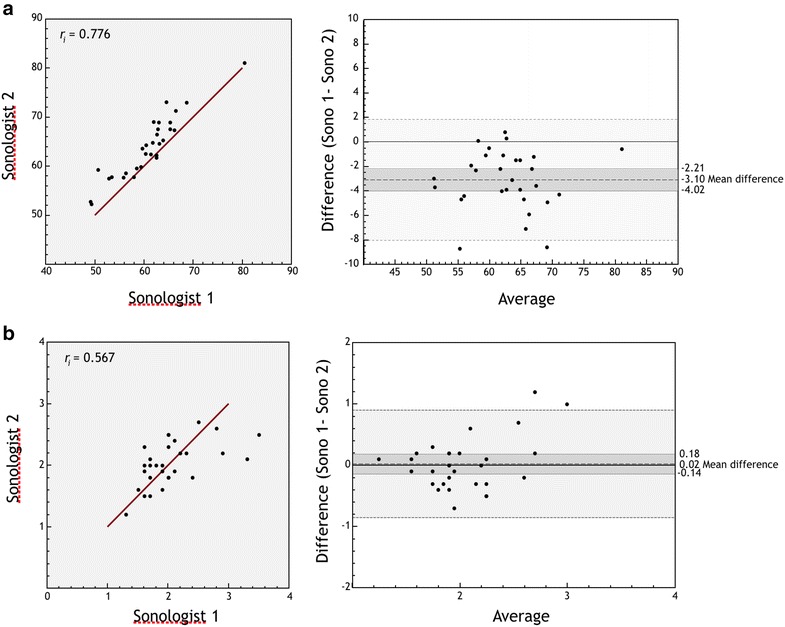
ICC graphs and Bland–Altman plots: agreement between sonologists in measurements. *Y* axis for Bland–Altman plots represents average of sonologists measurements. Mean difference between sonologists with 95% confidence intervals on the means shown (darker gray). Lighter gray bands represent the 95% confidence bands. **a** Skin tongue thickness (mm). **b** Epiglottis SA (mm)

**Fig. 4:**
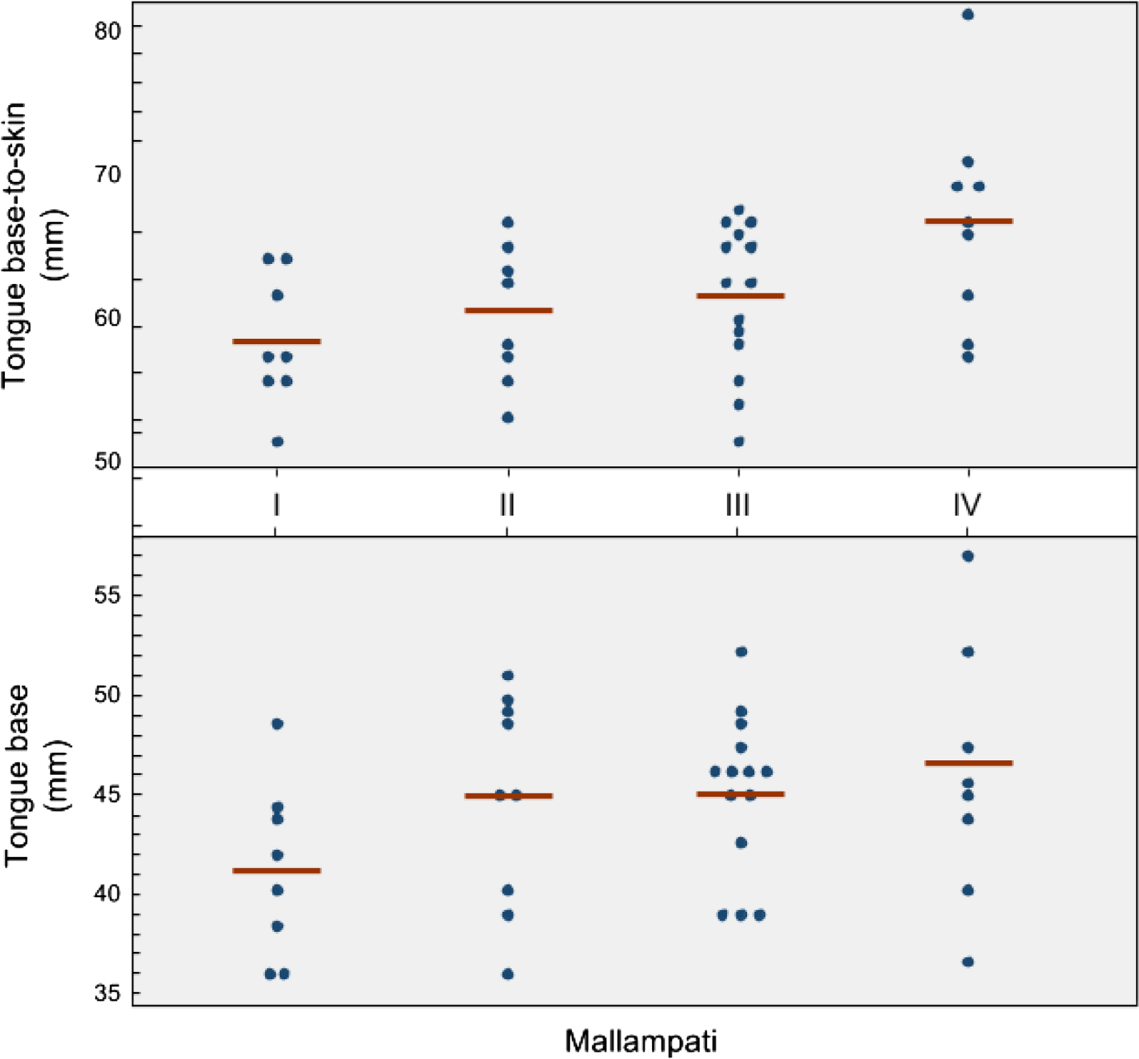
Tongue US measurements demonstrate linear increase as compared to Mallampati

Forty subjects were enrolled, of which one subject was excluded because epiglottic views were unobtainable, and in this patient, all other measurements were excluded for reasons of consistency. Of the 39 subjects with complete data, 31 were ED patients and 8 were volunteers: 50% were female, 50% white, 42% black, with median age 32.5 years (range 19–90) and BMI 26.0 (range 19–47). Mean ± SD for each measurement (mm) were as follows: tongue base (44.6 ± 5.1), tongue base-to-skin (60.9 ± 5.3), epiglottic width (15.0 ± 2.8) and thickness (2.0 ± 0.37), and pre-epiglottic space (11.4 ± 2.4). Intraclass correlation coefficients (ICCs) ranged from 0.76 to 0.88 for all measurements except epiglottis thickness (ICC = 0.57) and Bland–Altman plots demonstrated consistency over the range of values between the two physicians (Figs. 2, 3). Tongue base and tongue base-to-skin thickness were found to linearly increase with Mallampati score (*p* = .04, .01) (Fig. 4), whereas only tongue-to-skin thickness was loosely correlated with BMI (*r* = .38).

## Discussion

The study found that the airways of a randomly chosen population of ED patients with a range of BMI and Mallampati scores can be measured by emergency sonologists with good inter-operator reliability. This study, to our knowledge, is the first that documents the feasibility of these measurements made by clinician-performed ultrasonography in the emergency department environment. Only one subject in 40 had an airway parameter that could not be obtained by ultrasound. Almost all of the measurements used including tongue base, tongue base-to-skin, epiglottic width, and pre-epiglottic space had fair to good ICCs ranging from 0.76 to 0.88. Epiglottic thickness, however, had a poor ICC at 0.57 which is related to the inherently small values of this structure, making standard errors in ultrasound measurements (often in the 1–2 mm range) mathematically much more impactful. This explanation is consistent with the finding that the ICC for epiglottic width (a larger distance) was similar to that of other parameters. Possibly using the ‘zoom’ mode on the ultrasound machine to enlarge the image might have improved the ICC for epiglottic thickness measurement; however, such maneuvers increase the complexity and time required for the task, both of which can be disadvantageous in the context of the time constraints inherent in emergency airway management [17].

Future studies comparing soft tissue measurements to metrics of airway difficulty such as the Cormack–Lehane grading system will reveal whether epiglottic thickness is necessary or even helpful in this setting. Our study also suggests that epiglottic thickness has little variation in the general population, which also makes it less likely to be a discriminatory metric. Of course such reasoning would not have any place in situations where acute epiglottic inflammation or edema are of concern, such as in cases of smoke inhalation, angioedema, or epiglottitis. Similarly, the absence of thickening might give some assurance in cases where epiglottic enlargement or obstruction is a concern.

There are several limitations in this study. Experienced airway sonologists are aware that differences in probe pressure applied while scanning the neck can significantly alter measurements of these superficial neck structures. It was thus emphasized during the ultrasound airway training that the sonologist place the probe against the neck with the least pressure needed to maintain skin contact. The goal of minimal probe pressure also coheres with the principle of avoiding anything that might narrow the upper airway in a dyspneic patient receiving preoxygenation.

The tongue is a dynamic muscle and its position within the mouth can significantly change its size and shape, and also possibly the thickness of the soft tissues of the hypopharynx [18, 19]. Patients were therefore asked to rest their tongue with the anterior tip touching the lower incisors. In unconscious, hypoxic, or altered patients, this would not be possible limiting the generalizability of the findings of the current study to the real clinical environment. We also chose to position patients’ supine with the neck extended, to ensure rigorous standardization. Since the “sniffing position” (neck flexed with the external auditory meatus level with the sternum and some head extension) is currently recommended for laryngoscopy, this also limits the generalizability of our study in the clinical environment. We believe that future studies might want to use the “sniffing position” for purposes of standardization, with the additional advantage that it would allow the ultrasound to be performed while the patient is being set up for intubation. Another limitation of the study is its failure to obtain data regarding the time needed to perform the ultrasound exam. In the context of emergent airway management, any ultrasound requiring much more than 30–60 s would be unlikely to see widespread acceptance by clinicians unless the information it provided was of critical importance. The time taken to perform ultrasound assessment will need to be the subject of future inquiry, but in the current feasibility paper the time required for ultrasound assessment was not recorded because it was felt that any information obtained would not be valid in a real clinical context. Along these lines, it would be ideal if a single measurement was found to be predictive of difficult laryngoscopy, thereby obviating the need for the multiple measurements made in the current study. The study tried to correlate the ultrasound findings with established metrics used in pre-intubation airway assessment, but for reasons of patient comfort and practicability, some metrics were not included, such as hyoid-mental distance. This metric was not included because it was thought that it would likely be used in a pre-intubation airway evaluation regardless of whether or not an ultrasound was performed because it can be obtained without an ultrasound machine. Future studies might investigate this issue.

Finally, the ultrasound exams performed in the current study were done by advanced emergency sonologists receiving fellowship training. This will limit the generalizability of the study among emergency physicians, although it should be generalizable among anesthesiologists and critical care physicians with ultrasound experience and interest in the airway.

The tongue base and tongue base-to-skin measurements were found to correlate with increasing Mallampati score. This finding is not surprising given that Mallampati score is based on oropharyngeal anatomy, and it makes these ultrasound measurements less valuable, since the Mallampati can be obtained rapidly by visual inspection. Furthermore, the wide range of measured mean soft tissue thickness within each Mallampati grade makes it unlikely that ultrasound will be a useful substitute for the Mallampati score. In fact, one study found that combining Mallampati score with sonographic assessment of the skin to epiglottis distance was a stronger predictor of difficult airway compared to individual parameters [20]. Tongue base-to-skin thickness was the only measurement to loosely correlate with BMI [3, 5]. We expected this measurement to have a stronger relationship with BMI. Regardless, with respect to airway management previous research has demonstrated that large neck circumference is a more reliable predictor of difficult laryngoscopy than Mallampati, although also not without limitations [2, 21–23]. One study demonstrated that ratio of tongue thickness to thyromental distance was an independent predictor of difficult airway in patients undergoing anesthesia [24]. Since Cormack–Lehane grading can only be performed after intubation is under way, it would be of interest in future studies to determine whether pre-intubation ultrasound measurements of the pre-epiglottic space are predictive of CLG. Future studies would also need to assess whether the allocation of time and resources for ultrasonography during the fraught period prior to an emergency intubation are warranted by the additional information it generates. For purposes of expediency a single parameter would be ideal. In this context, pre-epiglottic distance appeared to be a potentially useful metric since it was measured with high ICC. It also has the advantage that the normal range of values that we measured was relatively wide: a standard deviation of 2.4 mm from the mean value of 11.4 mm gives an SD of 21% of the mean value. This means that standard errors of measurement are likely to have less impact and that real differences are likely to be detected. Additional investigation using a defined and standardized scanning method in pre-operative patients undergoing intubation is needed to determine whether ultrasound can serve as a non-invasive real-time predictor of difficult intubation.

## Conclusions

The current study demonstrates that emergency sonologists are able to obtain ultrasound measurements of the upper airway with a high degree of inter-operator reliability in a cross-section of undifferentiated patients and volunteers in the ED. Future studies are needed to determine whether similar accuracy is possible among patients receiving acute airway management in the ED; whether they are predictive of a difficult airway, and whether ultrasound measurements are sufficiently valuable to warrant the expenditure of time and resources needed to obtain them.

## Abbreviations

MS: Mallampati score
BMI: body mass index
ED: Emergency Department
EM: Emergency Medicine
SD: standard deviation
ICC: intraclass correlation coefficient
CLG: Cormack–Lehane Grade
